# Functional Role of Mitochondrial DNA in Cancer Progression

**DOI:** 10.3390/ijms23031659

**Published:** 2022-01-31

**Authors:** Yang-Hsiang Lin, Siew-Na Lim, Cheng-Yi Chen, Hsiang-Cheng Chi, Chau-Ting Yeh, Wey-Ran Lin

**Affiliations:** 1 Liver Research Center, Linkou Chang Gung Memorial Hospital, Taoyuan 333, Taiwan; yhlin0621@cgmh.org.tw; 2 Department of Neurology, Linkou Chang Gung Memorial Hospital, Taoyuan 333, Taiwan; siewna.lim@gmail.com; 3 College of Medicine, Chang Gung University, Taoyuan 333, Taiwan; 4 Department of Cell Biology and Anatomy, College of Medicine, National Cheng Kung University, Tainan 70101, Taiwan; cychen@gs.ncku.edu.tw; 5 Graduate Institute of Integrated Medicine, China Medical University, Taichung 404, Taiwan; hcchi@cmu.edu.tw; 6 Chinese Medicine Research Center, China Medical University, Taichung 404, Taiwan; 7 Department of Hepatology and Gastroenterology, Linkou Chang Gung Memorial Hospital, Taoyuan 333, Taiwan; 8 Molecular Medicine Research Center, Chang Gung University, Taoyuan 333, Taiwan

**Keywords:** mitochondria, SNP, mutation, ncRNA, prognostic marker, cancer

## Abstract

Mitochondrial DNA (mtDNA) has been identified as a significant genetic biomarker in disease, cancer and evolution. Mitochondria function as modulators for regulating cellular metabolism. In the clinic, mtDNA variations (mutations/single nucleotide polymorphisms) and dysregulation of mitochondria-encoded genes are associated with survival outcomes among cancer patients. On the other hand, nuclear-encoded genes have been found to regulate mitochondria-encoded gene expression, in turn regulating mitochondrial homeostasis. These observations suggest that the crosstalk between the nuclear genome and mitochondrial genome is important for cellular function. Therefore, this review summarizes the significant mechanisms and functional roles of mtDNA variations (DNA level) and mtDNA-encoded genes (RNA and protein levels) in cancers and discusses new mechanisms of crosstalk between mtDNA and the nuclear genome.

## 1. Introduction

**Figure 1:**
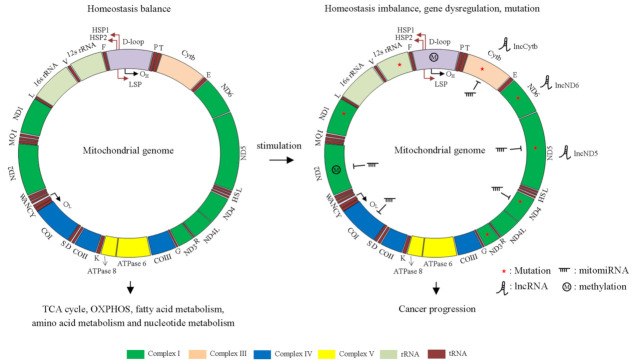
Overview of the human mitochondrial genome, including protein-coding genes, noncoding RNAs and control regions. The mtDNA is double-stranded and circular, with approximately 16,569 base pairs, which encode 2 rRNAs, 22 tRNAs and 13 mitochondrial protein subunits. The rRNA genes are in teal. Complex I genes are in green. Complex III genes are in peach buff. Complex IV genes are in blue. Complex V genes are in yellow. The regulatory region, D-loop, is shown in amethyst. Two promoters in mtDNA, heavy strand promoters (HSP) and light strand promoters (LSP), are shown. **Left panel**: Normal mitochondria functions as regulators for maintaining cellular homeostasis, such as the TCA cycle, OXPHOS and fatty acid metabolism. **Right panel**: Mitochondrial DNA mutations (red star), mitochondrial genes dysfunction and methylation lead to homeostasis imbalance and cancer progression. F: tRNA Phe, V: tRNA Val, L: tRNA Leu, I: tRNA Ile, Q: tRNA Gln, M: tRNA Met, W: tRNA Trp, A: tRNA Ala, N: tRNA Asn, C: tRNA Cys, Y: tRNA Tyr, S: tRNA Ser, D: tRNA Asp, K: tRNA Lys, G: tRNA Gly, R: tRNA Arg, H: tRNA His, S: tRNA Ser, L: tRNA Leu, E: tRNA Glu, T: tRNA Thr and P: tRNA Pro.

The sequence of the mitochondrial genome was first identified in 1981 [1]. Mitochondria contain distinct cell membranes and their own genome (circular DNA), which encode 2 rRNAs, 22 tRNAs and 13 mitochondrial protein subunits [2]. Mitochondria function as modulators for regulating cellular metabolism, including the tricarboxylic acid (TCA) cycle, oxidative phosphorylation (OXPHOS), fatty acid metabolism, amino acid metabolism and nucleotide metabolism [3,4]. Adenosine triphosphate (ATP) is the major energy source for maintaining cellular function. ATP is produced by glycolysis and OXPHOS. ATP is quickly generated by glycolysis and supports cancer cell survival, drug resistance and tumor metastasis [5]. In contrast, OXPHOS can produce 36 ATPs per glucose. Due to high glycolysis utilization in cancer cells, OXPHOS is downregulated in several cancer types [6], and several studies have found that OXPHOS is also upregulated in some cancers [6]. These findings suggest that OXPHOS plays dual roles in cancer progression. An imbalance in mitochondrial homeostasis causes excessive reactive oxygen species (ROS) production, which leads to DNA damage, apoptosis, aging and cancer progression [7,8]. To prevent excessive ROS, cells maintain homeostasis through the activation of the antioxidant reaction. Manganese superoxide dismutase (MnSOD) acts as the scavenger to eliminate ROS produced from mitochondria [9]. In the current central dogma of biology, it is well-known that DNA contains the important genetic information for making RNA (transcription), and then, RNA makes proteins (translation). Subsequently, proteins are responsible for regulating multiple cellular functions. In addition, a kind of gene, referred to as a noncoding gene, can be transcribed to RNA, but it cannot translate to a protein. Increasing evidence supports that noncoding RNA (ncRNA) also play an important role in modulating cell growth, metastasis, cell metabolism and mitochondria homeostasis [10]. Functional and clinical studies have reported that mitochondrial DNA (mtDNA) mutations, mtDNA single-nucleotide polymorphisms (SNPs), mtDNA-encoded microRNAs (mitomiRs), mitochondria-derived long noncoding RNAs (lncRNAs) and mitochondrial proteins are involved in cancer progression (Figure 1). Furthermore, nuclear-encoded genes, including transcription factors and ncRNAs, regulate mitochondria-related gene expression and homeostasis. Alterations in nuclear DNA-encoded genes lead to an imbalance in mitochondrial homeostasis, suggesting that crosstalk between the nuclear and mitochondrial genomes is important in cancer biology. Taken together, these findings suggest that mitochondria-related molecule (DNA, RNA and protein) is crucial for several important physiological homeostasis and cancer progression. Therefore, this review summarizes the functions and clinical relevance of the mitochondrial DNA (DNA level), mitochondria-encoded ncRNA (RNA level) and proteins (protein level) in cancer progression and discusses new mechanisms of crosstalk between mtDNA and the nuclear genome.

## 2. The Genetic Information of mtDNA and Nuclear DNA on Cancer Progression

Increasing evidence indicates that DNA mutation, SNP or gene expression act as a useful prognostic marker in cancer formation [11,12,13]. Here, we summarize the mtDNA/nuclear DNA variations and mitochondrial/nuclear gene expression on cancer progression.

### 2.1. The Role of mtDNA (SNP/Mutation) in Cancer

In fact, tumor cells can reprogram bioenergetic signal transduction, such as glucose metabolism and ROS production, to support survival [14,15]. On the other hand, mtDNA variations have been confirmed to be correlated with cancer progression. Jin et al. [16] investigated the association between mitochondrial ND3 SNPs and gastric cancer development. Three SNPs (rs28358278, rs2853826 and rs41467651) of ND3 were significantly correlated with the risk of gastric cancer. mtDNA mutations in the ND1 gene (G3842A), ND4 gene (A11708G) and ND5 gene (12418insA) were found to be correlated with hepatocellular carcinoma (HCC) progression [17]. In addition, Akouchekian and co-workers demonstrated that MT-ND1 gene mutation (T4216C) was associated with colorectal cancer (CRC) progression [18]. Previously, MT-ND2 mutation (G4776A) enhanced the cell growth of head and neck cancer cell via the induction of HIF1α [19]. Furthermore, this mutation enhanced pyruvate dehydrogenase kinase 2 expression through the induction of ROS production. Another report demonstrated that the impairment of mitochondrial complex I activity caused by mtDNA mutation was associated with the cisplatin-resistant phenotype. Based on a structural analysis, MT-ND2 mutation (T4587C) may play an important effector for promoting drug resistance [20]. Ishikawa and co-workers demonstrated that the MT-ND6 insertion mutation (13885insC) suppressed complex I activity and induced ROS production, resulting in the induction of cell metastasis in lung and breast cancer cells [21,22]. Another mutation in MT-ND6 (C12084T) was identified and correlated with metastatic capacity in a breast cancer cell line [23]. A previous study indicated that SNPs in MT-ND1 (T3394C and C3497T) were correlated with distant metastasis [24]. Accordingly, OXPHOS-related genes are important in regulating cancer cell metastasis. Kim et al. demonstrated that SNPs in uncoupling protein 2 (UCP2) and UCP3 genes are associated with mitochondria-related metabolism and healthy aging in females [25]. These findings suggest that significant variations in the corresponding genes may interfere with their own gene expression, subsequently disrupting cellular homeostasis and contributing to cancer formation. Shidara et al. demonstrated that ectopic expression of ATP6 mutation (T8993G or T9176C) induced tumor growth through inhibition of apoptosis [26]. A previous meta-analysis found that mutations in mitochondrial tRNA genes have also been linked to cancer progression. The study indicated that five tRNA mutations (mt-tRNA^Ala^, mt-tRNA^Arg^, mt-tRNA^Leu^, mt-tRNA^Ser^ and mt-tRNA^Thr^) were rarely found in healthy specimens [27]. These mutations may affect tRNA metabolism in lung cancer. In addition, Meng et al. [28] reported that tRNA mutations (mt-tRNA^ASP^) caused their tertiary structure and led to impairment of mitochondrial protein synthesis in breast cancer. Two noncoding genes (MT-RNR1 and MT-RNR2) are encoded by mitochondrial DNA and responsible for mitochondrial protein synthesis. Notably, SNPs in those noncoding regions were found to act as prognostic markers. Our group demonstrated that MT-RNR1 was significantly correlated with the survival outcomes of patients with HCC [29]. In addition, the expression levels of the glycolytic regulator hexokinase 2 (HK2) were higher in the MT-RNR1 709A group than in the MT-RNR1 709G group. Notably, the combined effects of MT-RNR1 709A and HK2 on survival outcome in HCC patients were poor. Taken together, we found that MT-RNR1 709A acted as an independent risk factor for overall survival and metastasis-free survival. Another study indicated that MT-RNR1 mutations (652G insertion and 716G) were identified only in gastric cancerous tissues, especially in intestinal type gastric cancer [30], suggesting MT-RNR1 mutations were correlated with incidence of intestinal type gastric cancer. Therefore, the mtDNA SNP/mutation may cause nonsense, missense or frameshift mutation, leading to loss of protein function or alteration of RNA structure and then modulated cancer progression.

### 2.2. The Association between Mitochondrial-Related Noncoding RNA and Cancer Progression

**Figure 2:**
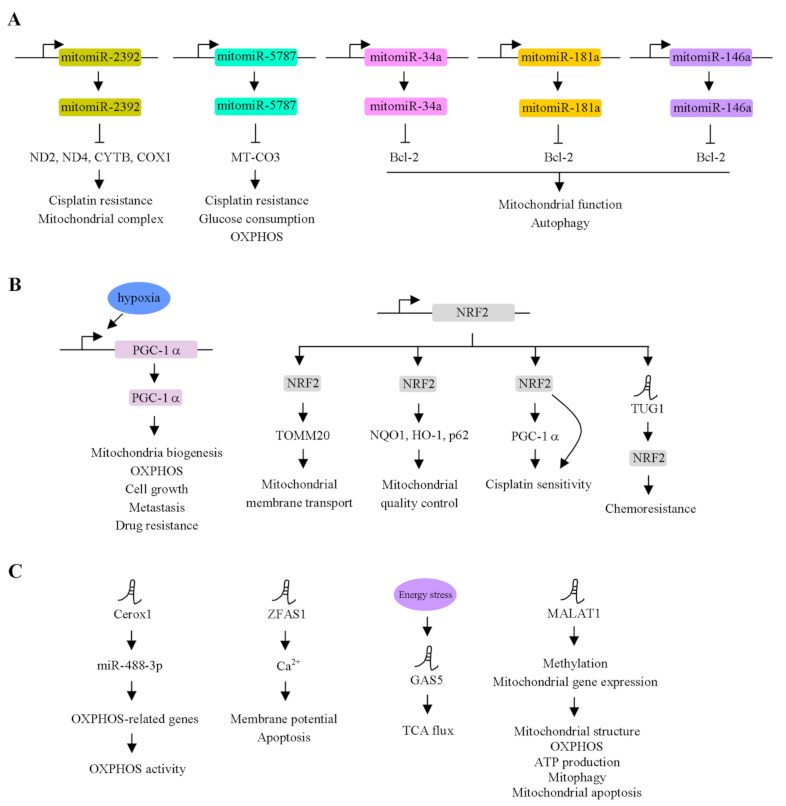
Functional roles of mtDNA-encoded miRNAs and nuclear DNA-encoded genes in cancer progression. (**A**) mtDNA-encoded miRNAs, including mitomiR-2392, mitomiR-5787, mitomiR-34a, mitomiR-181a and mitomiR-146a, inhibited the corresponding target genes and contributed to modulate drug resistance, mitochondrial functions, OXPHOS and autophagy. (**B**) Nuclear DNA-encoded protein coding genes, PGC-1α and NRF2, regulated the mitochondrial functions, cell growth, metastasis and drug resistance via modulation different downstream effectors. (**C**) Nuclear DNA-encoded lncRNAs regulate the mitochondria-related functions via modulation of downstream gene expression.

Recently, ncRNAs have been identified by next-generation sequencing (NGS) and act as crucial regulators of cancer progression. Depending on the length of ncRNAs, ncRNAs can be divided into two categories. One is small ncRNAs. The other is long ncRNAs. In fact, several different small ncRNAs have been identified. Of these, microRNAs (miRNAs) are involved in regulating cellular function via modulation of their target gene expression [31]. Notably, mtDNA-encoded miRNAs have been described. The term mitomiRs indicates that these miRNAs are mainly expressed in mitochondria [3]. Previous evidence reported that mitomiRs targeted nuclear- or mitochondrial-encoded mRNA and suppressed target gene expression, leading to the regulation of mitochondrial homeostasis (Figure 2A). The mRNAs encoded by the mitochondrial genome without or with short 3′UTRs may cause miRNA-RISC to not directly interact with target genes. Accordingly, the regulatory mechanism of mitomiR and its target gene remain unclear. Overexpression of mitomiR-2392 has been shown to induce cisplatin resistance through modulation of mitochondrial complex activity [32]. The results obtained in vitro were similar to those *in vivo*. Furthermore, several mitochondrial genes, such as ND2, ND4, ND5, CYTB and COX1, were regulated by mitomiR-2392 in an AGO2-dependent manner. In the clinic, mitomiR-2392 was negatively correlated with chemosensitivity and overall survival. The same group provided additional evidence to support that mitomiR is involved in cellular functions [33]. They found that mitomiR-5787 expression levels were increased in cisplatin-resistant tongue squamous cell carcinoma (TSCC) cells. Knockdown of mitomiR-5787 repressed MT-CO3 translation. Functionally, mitomiR-5787 regulates cisplatin resistance, glucose consumption and OXPHOS in TSCC cells. Giuliani et al. [34] demonstrated that mitomiR-34a, -181a and 146a were highly expressed in replicative senescent human umbilical vein endothelial cells (HUVECs) compared to younger HUVECs. In senescent HUVECs, these mitomiRs regulated mitochondrial function and autophagy through modulation of Bcl-2. Taken together, these mitomiRs are mainly expressed in mitochondria and responsible for regulating cell growth, drug resistance and apoptosis.

LncRNAs can associate with proteins and form a complex to regulate target gene expression by different mechanisms, such as miRNA sponges, recruitment of chromatin modifiers and regulation of mRNA splicing, stability and translation [35]. Emerging evidence has shown that lncRNAs can be encoded from the mitochondrial genome. Rackham and colleagues demonstrated that mitochondrial long ncRNAs, including lncND5, lncND6 and lncCyt b RNA, were identified by deep sequencing [36]. Specifically, lncND5 RNA levels were highly abundant compared to lncND6 and lncCyt b RNA levels. The three lncRNAs were regulated by nuclear-encoded proteins [37]. Another report indicated that the lncND5 transcript was positively regulated by pentatricopeptide repeat domain (PTCD) 1 [38]. On the other hand, depletion of PTCD2 repressed lncND5 and lncND6 expression [38]. In addition, a nuclear DNA-encoded protein, mitochondrial RNase P protein 3, localized in mitochondria regulated lncND5, lncND6 and lncCyt b RNA abundance [37]. A lncRNA named long intergenic noncoding RNA predicting cardiac remodeling (LIPCAR) is a chimeric fusion transcript between the 5′-end of COX2 and the 3′ end of CYTB [39,40]. Furthermore, LIPCAR served as a novel biomarker for predicting survival outcomes in patients with heart failure. Therefore, lncRNAs encoded from mitochondria genome is involved in regulating cellular homeostasis and cancer progression.

### 2.3. Dysregulation of Mitochondrial-Encoded Protein-Coding Genes in Cancer Progression

As mentioned above, several genes are encoded by mitochondria genome. In this section, we summarize the association between mitochondrial-related proteins and cancer progression. A previous study showed that MT-ND2 expression was increased in colorectal cancer samples compared to corresponding noncancerous tissues [41]. MT-ND2 expression in Caco-2 cells was upregulated by treatment with a demethylation drug, 5-aza-2′deoxycytidine (5-Aza). Notably, methylation status in the D-loop region was found to be associated with MT-ND2 expression. Liu et al. demonstrated that highly methylated D-loop region of mtDNA was observed in bone metastatic tumor cells from renal cell carcinoma (RCC) compared to those in the primary RCC tissues [42]. In addition, mRNA levels of MT-ND2, MT-ND3, MT-ND4L, MT-ND6, ATP6, ATP8, COI and COII were significantly downregulated in bone metastatic tumor cells derived from RCC compared to those in parental RCC cells. Notably, 5-Aza treatment contributed to repression of tumor metastasis. These observations suggested that mitochondrial gene expression was controlled by epigenetic regulation. Li and co-workers demonstrated that MT-ND5 and MT-ND6 expression was inversely associated with tumor stage in lung squamous cell carcinoma (LUSC) and lung adenocarcinoma (LUAD) [43]. Furthermore, in the Cox regression analysis, a lower expression of these two genes was significantly associated with shorter survival outcomes. A group demonstrated that the overexpression of cytochrome B (Cytb) mutation (mtCytb) in MB49 bladder cancer cell lines promoted ROS production, oxygen utilization and lactate production, which led to tumor growth, metastasis and angiogenesis induction in vitro and in vivo [44]. Mechanistically, mtCytb stimulated the nuclear factor-κB2 (NF-κB2) signaling pathway. These findings supported that mitochondrial-encoded protein mutations (i.e., Cytb mutation) played an oncogenic role in a bladder cancer cell line. Notably, OXPHOS was regulated by mitochondrial ATP synthase [45]. Direct or indirect inhibition of ATP synthase by inhibitors resulted in the repression of cell growth in multiple cancer cell lines [46]. Compared with adjacent normal tissues, the expression levels of MT-COI, MT-CYB, MT-ND1 and MT-RNR1 in CRC adenomas and adenocarcinomas were progressively increased using a quantitative real-time PCR analysis [47]. Therefore, the dysregulation of mitochondria-encoded genes contributes to cancer progression.

## 3. Nuclear DNA-Encoded Genes Function as Modulators to Coordinate Mitochondrial Gene Expression and Mitochondrial Functions

Accumulating evidence indicates that nuclear DNA-encoded genes serve as a component of OXPHOS or leads to regulating mitochondria homeostasis via the modulation of mitochondria-related gene expression [48]. These findings suggest that both of them are a key effector for maintaining cellular functions. Accordingly, we summarize how nuclear gene-encoded transcription factors crosstalk with mitochondrial gene expression (Figure 2B,C).

### 3.1. Action of Peroxisome Proliferator-Activated Receptor-γ Coactivatior-1α (PGC-1α) in Mitochondria

PGC-1α functions as a transcription factor for modulating mitochondrial biogenesis, gluconeogenesis and OXPHOS [49,50,51]. LeBleu et al. demonstrated that the transcription coactivator PGC-1α is involved in mitochondrial biogenesis and OXPHOS in an invasive cell line [52]. The expression level of the α subunit of ATP synthase was upregulated by PGC-1α in 4T1 cells. In contrast, the α subunit of ATP synthase was downregulated in prostate cancer and correlated with earlier-onset prostate cancer [53]. On the other hand, the β subunit of ATP synthase was expressed at lower levels in hepatoma, colon cancer and LUAD [54,55]. Another report showed that hypermethylation in the promoter of the ATP5B gene was found, thereby silencing its gene expression [56]. A previous study indicated that hypoxia induced cell growth, metastasis and mitochondrial function by the promotion of PGC-1α expression in CRC cell lines [57]. Moreover, the overexpression of PGC-1α in hypoxia abolished the 5-fluorouracil-induced CRC cell line by apoptosis. These findings suggest that nuclear gene-encoded PGC-1α is responsible for regulating mitochondrial gene expression and cell migration.

### 3.2. Action of NF-E2-Related Factor 2 (NRF2) in Mitochondria

Similar to PGC-1α, the nuclear DNA-encoded gene NF-E2-related factor 2 (NRF2) is an important transcription factor for regulating nuclear and mitochondrial gene expression, which are involved in the electron transfer chain (ETC) reaction [58,59,60]. Under normal physiological conditions, NRF2 is regulated by the proteasome degradation pathway via the Kelch-like ECH-associated protein 1 (Keap1)-Cul3 E3 ligase. Keap1 is oxidized by ROS, resulting in the promotion of NRF2 expression [61]. Subsequently, NRF2 can translocate into the nucleus and associate with the Maf protein to bind to the antioxidant response element (ARE; 5′-TGACNNNGC-3′) of the target genes, in turn promoting their expression [62]. Heiss et al. demonstrated that the activation of NRF2 induced glucose utilization in fibroblasts. Moreover, alteration of the glucose concentration led to the repression of NRF2-mediated detoxication. NRF2 modulates the pentose phosphate pathway through the regulation of glucose 6-phosphate dehydrogenase expression, resulting in the regulation of nucleotide and NADPH production [63]. A previous study [64] indicated that NAD(P)H quinone oxidoreductase (NQO1), heme oxygenase-1 (HO-1) and p62 genes involved in mitochondrial quality control were regulated by NRF2. Previously, the overexpression of NRF2 contributed to induction of the oxygen consumption rate, higher basal ATP levels and higher basal mitochondrial membrane potential [65]. Transcription factor A, mitochondria (TFAM), transcription factor B1, mitochondria (TFB1M) and TFB2M are regulated by NRF1 [66]. Additionally, translocase outer mitochondrial membrane (TOMM) 20, responsible for modulating mitochondrial membrane transport, was also regulated by NRF2 [67]. Increasing evidence indicates that mitochondrial biogenesis and gene expression are tightly regulated by the PGC-1α/NRF1/NRF2 axis [68]. Deng et al. demonstrated that PGC-1α modulated NRF2-mediated functions via the regulation of GSK3β in the ovarian cancer cell line SKOV3, which is resistant to platinum [69]. Moreover, PGC-1α is directly regulated by NRF2 at the transcriptional level [70]. Taken together, reciprocal regulation between PGC-1α and NRF2 was a pivotal effector for regulating mitochondrial functions.

### 3.3. Action of lncRNAs in Mitochondria

In this subsection, we summarize the association between nuclear-encoded lncRNAs and cancer progression. Two lncRNAs, cardiomyocyte-enriched noncoding transcript (Caren) and cardiac apoptosis-related lncRNA (CARL), are encoded by the nuclear genome and function as modulators for the regulation of mitochondrial biogenesis and fission [71,72]. An oncogenic lncRNA, taurine upregulated gene 1 (TUG1), had no effect on the NRF2 mRNA levels. Alternatively, NRF2 protein expression was dramatically regulated by TUG1 by interacting with NRF2, in turn conferring chemoresistance in esophageal carcinoma [73]. P32 functions as a master regulator to ordinate OXPHOS and glycolysis [74]. In melanoma, lncRNA SAMMSON is associated with p32 and results in the regulation of prooncogenic function [75]. Sirey and co-workers [76] demonstrated that Cerox1 was a cytoplasmic lncRNA and regulated OXPHOS-related gene expression, leading to modulating OXPHOS enzymatic activity. Furthermore, the effect of Cerox1 on mitochondria was mediated by miR-488-3p. Previous findings showed that the overexpression of ZFAS1 lncRNA reduced the mitochondria membrane potential and triggered the mitochondria apoptosis pathway through the alteration of Ca^2+^ homeostasis in cardiomyocyte [77]. Zhao et al. demonstrated that metastasis-associated lung adenocarcinoma transcript 1 (MALAT1) was highly expressed in the mitochondria of HCC cell lines [78]. Notably, MALAT1 associated with mitochondrial genes such as D-loop, ND3, COX2 and CYTB. The repression of MALAT1 modulated mtDNA methylation and mitochondrial gene expression, leading to alteration of the mitochondrial structure, OXPHOS, ATP production, mitophagy and mitochondrial apoptosis. Recently, growth arrest-specific 5 (GAS5) lncRNA has been found to be a tumor suppressor for modulating energy homeostasis in cells [79]. GAS5 was regulated upon energy stress. GAS5 modulated TCA flux by altering the association of components of the TCA cycle, including citrate synthase, fumarate hydratase and malate dehydrogenase. In the clinic, GAS5 expression was negatively correlated with those genes in breast cancer tissues. This finding suggests that nuclear DNA-encoded genes (lncRNAs and proteins) and mtDNA-encoded genes crosstalk together and then maintain cellular homeostasis.

## 4. Strategy for Investigating Mitochondrial Function in Cell Lines

The effects of mtDNA-mediated cellular functions have been well-documented, playing an important role in metabolism and energy production. More than a decade ago, long-term and low-dose EtBr treatment intercalated into circular DNA and repressed mtDNA replication and transcription without interfering with the corresponding nuclear DNA. Thus, to explore the importance of mitochondria in vitro, mitochondrial DNA-depleted cell lines (ρ^0^) were generated by ethidium bromide (EtBr) treatment in specific cell lines. Then, human subject mitochondria could be transferred into the ρ^0^ cell line, and the transferred cell line was called the cytoplasmic hybrid (cybrid) cell line. Notably, this model was used to investigate mitochondria-related cancers. However, the ρ^0^ cell lines were only generated in specific cell lines, such as cervical cancer (HeLa) and hepatoma (SK-Hep1) cell lines, because long-term EtBr treatment may cause DNA mutation and cell death. Alternatively, Correia-Melo et al. [80] designed another methodology to deplete mtDNA: that is, enforced mitophagy. Overexpression of the ubiquitin E3 ligase Parkin, combined with mitochondrial inhibitors carbonyl cyanide 3-chlorophenylhydeazone (CCCP), antimycin A and oligomycin A, induced mitophagy, thereby effectively depleting mitochondria in cell lines. This methodology maintained ρ^0^ cell lines for approximately one month with minimal cell cytotoxicity and long-term investigations. It is difficult to investigate the functional role of a single SNP or mutation in the mtDNA sequence, because mtDNA has a high copy number in the cell. A previous study [81] demonstrated that mtDNA levels and transcription were repressed by clustered regularly interspaced short palindromic repeats (CRISPR)-Cas9-mediated mtDNA cleavage. To avoid Cas9 ubiquitously expressed in the nucleus or mitochondria, the authors generated a Cas9—namely, mitoCas9—that was only localized in mitochondria. Then, this modification successfully reduced the mtDNA levels and transcription in the HEK293 cell line but not in nuclear DNA. These findings may raise an important question: Is it possible to study point mutations or SNPs of mtDNA by CRISPR-Cas9 that are involved in cancer progression? Enforced mitophagy and CRISPR-Cas9 are both novel methodologies for investigating mitochondrial importance in cancer biology. More successful mtDNA-depleted cell lines and studies to support those two methods are needed to observe the mitochondrial function.

## 5. Mitochondria Target Therapies and Their Application

Energy production and cellular functions are tightly controlled by mitochondria. The dysregulation of mitochondrial homeostasis may cause cancer progression. This is why mitochondria act as a target for cancer therapy. The different consequences of mitochondria-mediated cellular functions may be caused by different mtDNA SNPs, mutation sites and dysregulated gene-mediated mitochondrial function. Several reagents/inhibitors have been designed and tested to regulate mitochondrial homeostasis for cancer treatment. These agents are divided into different actions of mitochondrial therapy, including mitochondrial protection, biogenesis, quality control and signaling pathways. One type of molecule, delocalized lipophilic cations (DLCs), was designed and successfully accumulated in the mitochondrial matrix. Subsequently, one DLC, tetraphenylphosphonium (TPP), has been used to assist drug delivery to target mitochondria. Antioxidants such as lipoic acid and vitamin E conjugated with TPP preferentially accumulate in mitochondria [82,83]. Notably, the anticancer drug doxorubicin was also conjugated with TPP and applied for treating drug resistance in breast cancer [84]. They found that TPP-doxorubicin treatment caused more cell death than doxorubicin treatment. Although these findings indicated that positive and lipophilic molecules were useful for cancer treatment, the uptake of these positive molecules by cells was less specific. The consequences of these side effects may cause unexpected results.

Intracellular antioxidant coenzyme Q10 (CoQ10) is well-known for protecting against peroxidation-induced plasma membrane damage and has been applied for treating metabolic syndrome and type 2 diabetes patients [85,86]. A study demonstrated that a liposomal-based nanocarrier MITO-Porter was successfully designed and applied for cancer treatment [87,88]. This carrier delivers the drug into the mitochondria through membrane fusion. MITO-Porter associates with CoQ10, in turn inducing its accumulation in mitochondria. 5-Aminoimidazole-4-carboxamide ribotide (AICAR) regulated the ATP levels and suppressed ROS production. Additionally, AICAR promoted mitochondrial biogenesis but did not modulate the mitochondrial membrane potential. Many compounds have been designed and applied for targeting energy homeostasis via the modulation of AMP-activated protein kinase (AMPK), mammalian target of rapamycin (mTOR), NRF1 and TFAM [89].

## 6. Conclusions

Recently, mitochondrial homeostasis has become an important event for cancer progression. An increasing number of studies have found that mtDNA SNPs and mutations act as predictors or prognostic biomarkers in cancers. Notably, mtDNA-encoded genes and nuclear DNA-encoded genes tightly crosstalk and maintain cellular functions. The mtDNA SNPs (DNA level), mutations (DNA level), mtDNA-encoded genes (RNA and protein levels) and nuclear DNA-encoded gene (RNA and protein levels) networks modulating mitochondrial functions in cancers are comprehensively listed in Table 1 and Table 2. As mentioned previously, these studies focused on the functional roles of one specific target gene encoded by mtDNA or nuclear DNA. Now, the promising omics technology (from genomics to transcriptomics to proteomics to metabolomics) is crucial to investigate the mitochondria-associated cancer progression step by step. Eventually, integration with those results will be important for accurately identifying specific signaling pathways associated with mitochondrial dysfunction and developing the therapeutic interventions. In this review paper, we highlighted the functional roles of these phenomena associated with mitochondrial homeostasis and summarized their potential mechanisms.

## Figures and Tables

**Table 1:** The association between mtDNA SNP/mutations (DNA level) and cancer progression.

Gene Name	SNP/Mutation	Mediated-Cellular Functions	Cancer	Reference
ND1	G3842A	-	HCC	[17]
ND1	T4216C	-	CRC	[18]
ND1	T3394C	Metastasis	-	[24]
ND1	C3497T	Metastasis	-	[24]
ND2	G4776A	Cell growth and ROS production	Head and neck cancer	[19]
ND2	T4587C	Drug resistance and Mitochondrial complex I activity	RCC	[20]
ND3	rs28358278, rs2853826, and rs41467651	-	Gastric Cancer	[16]
ND4	A11708G	-	HCC	[17]
ND5	12418insA	-	HCC	[17]
ND6	13885insC	Complex I activity and ROS production	Lung and Breast Cancer	[21,22]
ND6	C12084T	Metastasis	Breast Cancer	[23]
UCP2	rs591758 and rs675547	-	-	[25]
UCP3	rs1626521	-	-	[25]
MT-ATP6	T8993G and T9176C	Cell growth and Apoptosis	Head and neck squamous cell carcinoma	[26]
MT-RNR1	G709A	Metastasis	HCC	[29]
MT-RNR1	652G insertion and 716G	-	Gastric cancer	[30]

**Table 2:** The actions of mitochondria-encoded and nuclear-encoded genes (RNA and protein level) in cancer progression.

Gene Name	Principal Functions	Molecules and Signaling Pathways Involved	Study Model	Prognostic Markers in Cancer	Cancer Development	Reference
mitomiR-2392	Cisplatin resistance, mitochondrial complex activity	ND2, ND4, ND5, CYTB, COX1	TSCC	✓	Progression	[32]
mitomiR-5787	Glucose metabolism, chemoresistance	MT-CO3	TSCC	✓	Repression	[33]
mitomiR-34a	Autophagy	Bcl-2	HUVEC	-	-	[34]
mitomiR-181a	Autophagy	Bcl-2	HUVEC	-	-	[34]
mitomiR-146a	Autophagy	Bcl-2	HUVEC	-	-	[34]
lncND5	Mitochondrial gene expression	PTCD1, mitochondria RNase P protein 3	-	-	-	[36,37]
lncND6	Mitochondrial gene expression	PTCD1, PTCD2, mitochondria RNase P protein 3	-	-	-	[36,37]
lncCytb	Mitochondrial gene expression	Mitochondria RNase P protein 3	-	-	-	[36,37]
LIPCAR	-	-	Heart failure	✓		[39]
ND2	Epigenetic regulation, mtDNA copy number	Methylation in D-loop	Colorectal cancer	-	Progression	[41]
ND5	-	-	LUSC, LUAD	✓	Repression	[43]
ND6	-	-	LUSC, LUAD	✓	Repression	[43]
CYTB	ROS production, oxygen utilization, lactate production, metastasis, angiogenesis	NFκB2 signaling pathway	Bladder cancer	-	Repression	[44]
PGC-1α	Mitochondria biogenesis, OXPHOS, metastasis	ATP synthase	Breast cancerColorectal cancer	✓	Progression	[52]
ATP5F1A	OXPHOS	-	Prostate cancer	✓	Progression	[53]
ATPase	HSP60	-	LUAD	✓	Repression	[56]
ATP5B	Hypermethylation, drug resistance	-	Chronic myeloid leukemia	-	-	[56]
NRF1	Metabolic homeostasis	TFAM, TFB1M, TFB2M	-	✓	-	[66]
NRF2	Oxygen consumption, ATP level, mitochondrial membrane potential, mitochondria membrane transport	TOMM20	Glial cells, mouse embryonic fibroblast	✓	Progression/Repression	[69]
lncRNA Caren	Mitochondrial biogenesis and fission	ATM/DDR pathway, Hint1 expression	Cardiomyocyte	-	-	[71]
lncRNA CARL	Mitochondrial fission and apoptosis	miR-539, PHB2	Cardiomyocyte	-	-	[72]
TUG1	Chemoresistance	NRF2 interaction	Esophageal carcinoma	-	-	[73]
SAMMSON	OXPHOS, glycolysis, survival	P32	Melanoma	✓	-	[75]
Cerox1	OXPHOS, enzymatic activity	OXPHOS-related genes expressions, miR-488-3p	Mouse Neuro-2a neuroblastoma cells, HEK293 cells	-	-	[76]
ZFAS1	Mitochondria membrane potential, mitochondria apoptosis	Ca^2+^ homeostasis	cardiomyocyte	-	-	[77]
GAS5	Energy stress, TCA flux	Citrate synthase, fumarate hydratase and malate dehydrogenase	breast cancer	✓	Repression	[79]
MALAT1	Metabolic reprogramming	D-loop, ND3, COX2, CYTB	HCC	✓	Progression	[78]

## Data Availability

Not applicable.
